# SEMdag: Fast learning of Directed Acyclic Graphs via node or layer ordering

**DOI:** 10.1371/journal.pone.0317283

**Published:** 2025-01-08

**Authors:** Mario Grassi, Barbara Tarantino

**Affiliations:** Department of Brain and Behavioral Sciences, University of Pavia, Pavia, Italy; Chinese Academy of Sciences, CHINA

## Abstract

A Directed Acyclic Graph (DAG) offers an easy approach to define causal structures among gathered nodes: causal linkages are represented by arrows between the variables, leading from cause to effect. Recently, industry and academics have paid close attention to DAG structure learning from observable data, and many techniques have been put out to address the problem. We provide a two-step approach, named SEMdag(), that can be used to quickly learn high-dimensional linear SEMs. It is included in the R package **SEMgraph** and employs a two-stage order-based search using previous knowledge (Knowledge-based, KB) or data-driven method (Bottom-up, BU), under the premise that a linear SEM with equal variance error terms is assumed. We evaluated our framework’s for finding plausible DAGs against six well-known causal discovery techniques (ARGES, GES, PC, LiNGAM, CAM, NOTEARS). We conducted a series of experiments using observed expression (or RNA-seq) data, taking into account a pair of training and testing datasets for four distinct diseases: Amyotrophic Lateral Sclerosis (ALS), Breast cancer (BRCA), Coronavirus disease (COVID-19) and ST-elevation myocardial infarction (STEMI). The results show that the SEMdag() procedure can recover a graph structure with good disease prediction performance evaluated by a conventional supervised learning algorithm (RF): in the scenario where the initial graph is sparse, the BU approach may be a better choice than the KB one; in the case where the graph is denser, both BU an KB report high performance, with highest score for KB approach based on topological layers. Besides its superior disease predictive performance compared to previous research, SEMdag() offers the user the flexibility to define distinct structure learning algorithms and can handle high dimensional issues with less computing load. SEMdag() function is implemented in the R package **SEMgraph**, easily available at https://CRAN.R-project.org/package=SEMgraph.

## Introduction

The two primary fields of causality research are causal inference and causal discovery. The former emphasizes testing causal knowledge directly from observable data. It is the process of evaluating whether an observed association actually reflects a cause-and-effect relationship. The latter aims to deduce causal structure from data. In other words, find a causal model that accurately reflects a dataset.

A formal representation of the interactions between the observable variables, such as a casual graph, is crucial for causal inference, or the process of quantifying the influence of a cause on its consequence. In a wide range of fields, such as genetics [1], finance [2], and social science [3], a Directed Acyclic Graph (DAG) offers an elegant way to describe directional or causal structures among collected nodes. Learning the DAG structures from observable data has received a lot of attention recently from both academia and business.

Structure learning is a model selection problem in which one estimates or learns a graph that best captures the dependence structure in a given data set [4]. DAG learning is well known to be computationally difficult, and several algorithms have been proposed to solve it, using one of three possible approaches: constraint-based algorithms [5], which use conditional independence tests to learn the dependence structure of the data; score-based algorithms [6, 7], which maximize some goodness-of-fit scores in the potential graph space; and hybrid algorithms, which combine both approaches [8, 9]. However, the majority of the aforementioned methods can only restore a DAG’s Markov equivalence class. Exact DAG recovery has recently received a lot of attention. It has been demonstrated that algorithms based on correct node ordering are capable of differentiating between various DAGs in the same equivalence class. This advantage is attributable to more data distributional assumptions than just conditional independence relations. Several forms of the order-based algorithms have been shown to be able to produce unique causal directions, and have received practical applications. By designing the function and noise, a group of functional causal models are proposed, such as linear model with equal error variances (EqVarDAG, [10]), linear model with non-Gaussian error (LiNGAM, [11]), non-linear model with Gaussian error (ANM, [12]), and causal additive model (CAM, [13]).

The main contribution of this article is the development of a two-step algorithm for learning high-dimensional sub-Gaussian linear SEMs with the same error variances [14], called SEMdag() and included in the R package **SEMgraph** [15]. First, a 1) a node (vertex) or layer (level) ordering of the p nodes is extracted and then 2) the DAG is estimated using penalized (L1) regressions [16]. The estimated linear order is determined by a priori graph topological vertex (TO) or level (TL) ordering, or by using a data-driven Bottom-up (BU) approach. To investigate the utility of our approach, we used a training dataset for model training and a test dataset for evaluating classification performance. We performed four sets of experiments on Amyotrophic Lateral Sclerosis (ALS), Breast cancer (BRCA), Coronavirus disease (COVID-19) and ST-elevation myocardial infarction (STEMI). We tested the ability of our framework to discover plausible DAGs against six popular causal discovery methods, i.e. PC [5], GES [6], ARGES [9], directLINGAM [17], CAM [13], NOTEARS [18] to provide a meaningful comparison in terms of disease predictive performance.

The outline of the paper is as follows. Firstly, the problem setting is discussed, introducing different classes of structure learning methods and, in the end, our contribution. Then, the experimental design and the evaluation scheme is described. Finally, we present the main findings and a brief concluding discussion.

## Materials and methods

### Graphical and structural equation models

A DAG is defined as *G* = (*V*, *E*), where *V* is the vertex set and *E* is the set of directed edges. When there is an edge (*j*, *k*) ∈ *E*, the edge *k* → *j* is implied. The parent set and the set of children of the *j*-th node in the graph *G* are indicated, respectively, by the symbols *pa*(*j*) and *sib*(*j*). If *pa*(*j*) = ∅, the vertex *j* is a source (root) vertex in *G*; if *sib*(*j*) = ∅, the vertex *j* is a sink (leaf) vertex in *G*, otherwise the vertex *j* is a connector vertex in *G*.

If each variable in the child set can be expressed as a linear combination of the variables in its parent set, the system of linear equation represents a Structural Equation Model (SEM) as follows:

$$\begin{array}{c} Y_{j}=\sum_{k\in pa(j)}\beta_{jk}Y_{k}+U_{j},\;\;\;\;\;\;j\in V \end{array}$$ (1)

where *Y*_*j*_ and *U*_*j*_ are an observed variable and an unobserved (hidden) error term, respectively, while *β*_*jk*_ is a regression (path) coefficient. The error terms *U*_1_, …, *U*_*p*_ are independent with Gaussian distribution, *U*_*j*_ ∼ *N*(0, *σ*_*j*_), *j* ∈ *V*.

As a result, the joint distribution of *Y* factorizes according to the following decomposition of the DAG, *G*: $P\left(Y\right)=\prod_{j=1}^{p}P\left(Y_{j}|pa\left(j\right)\right)$. *P* is then called *Markov* w.r.t. *G*. Various assumptions for the model defined in Eq 1 are specified:

- *Causal sufficiency*: The absence of hidden (or latent) variables is referred to as causal sufficiency [5]. For modeling hidden variables, there are two typical approaches: (i) they may appear as a dependence between the error terms, *U* or (ii) they may be explicitly modeled as nodes in the structural equations. The absence of latent confounding in Eq (1) uses (i): the *U* terms are considered to be independent, i.e., cov(*U*_*j*_; *U*_*k*_) = 0 for all pairwise (*j*, *k*).
- *Causal faithfulness*: If there are no Conditional Independence (CI) relations other than those implied by the Markov property, the distribution of *P*(*Y*), produced by Eq (1), is faithful to a DAG *G*. This indicates that using the so-called d-separation rule [5], all CI can be read out from a DAG *G* if the distribution *P* is faithful to the DAG *G*. Given a set *S*, two nodes (*k*, *j*) are said to be d-separated if the conditional correlation between node *j* and *k* (given *S*) is equal to 0.
- *Acyclicity*: The DAG *G* needs to be acyclic, which implies that it is not feasible to start at any variable in the DAG, go ahead along the directed arrows, and then return to the same variable. Solution of the structural equations requires that (*I* − *B*) is invertible and can be interpreted as an instantaneous feedback system that converges to a stable equilibrium.
- *Linearity and Gaussianity*: Nodes (observed variables) of the DAG *G* can be expressed as a linear combination of its parents plus independent the Gaussian noise random variables, *U* ∼ *N*_*p*_(0, *D*_*σ*^2^_).

The different algorithm are discussed below, and their assumptions are summarized in Table 1.

**Table 1:** The assumptions of the considered structure learning methods.

Method	Causal faithfulness	Causal sufficiency	Graph acyclicity	Model linearity	Gaussian error	Equal error variances
PC	yes	yes	yes	yes	yes	no
GES	yes	yes	yes	yes	yes	no
ARGES	yes	yes	yes	yes	yes	no
LiNGAM	no	yes	yes	yes	no	no
CAM	no	yes	yes	no	ni	no
NOTEARS	no	yes	yes	ni	yes	yes
SEMdag	no	yes	yes	yes	yes	yes

### Structure learning methods

The problem of learning the structure of a SEM is as follows. Given an *n* × *p* data matrix, *Y* ≔ (*Y*_1_, …, *Y*_*p*_) with i.i.d *n* rows drawn from *G* and a SEM $\left(B,\left\{\sigma_{i}^{2}\right\}\right)$, we want to learn a $\hat{G}$ and a SEM $\left(\hat{B},\left\{{\hat{\sigma }}_{i}^{2}\right\}\right)$ from *Y* such that $G=\hat{G}$.

There are many structure learning techniques in use, of which we consider three broad approaches: CPDAG-based, order-based and gradient-based methods.

#### CPDAG-based methods

*G* is typically not identifiable from the distribution of *Y*, but we may determine its Markov equivalence class, or in other words, its Completed Partially Directed Acyclic Graph (CPDAG). Markov-equivalent DAGs have the same skeleton and v-structures [19, 20]. A v-structure consists of the triple *i* → *j* ← *k*, where *i* and *k* are not adjacent. Each Markov equivalence class may be represented as a CPDAG that can include both directed and undirected edges [21]. Only when the edge *j* → *k* is shared by all DAGs in the equivalence class, a CPDAG has the edge *j* → *k*. If a DAG with *j* → *k* and a DAG with *k* ← *k* are both present in the class, hence the CPDAG has the undirected *j* − *k*.

To learn the CPDAG (assuming causal faithfulness) the structure learning techniques may be divided into three classes [5, 22].

(1) *Constraint-based methods.* The constraint-based approach [5, 23] tests pairwise causal links using a local conditional independence criterion.

The PC algorithm [5] carries the names of its creators, Peter Spirtes and Clark Glymour. In order to understand the structure of the underlying DAG, it does a number of conditional independence tests. In particular, it learns the CPDAG of the underlying DAG in three steps that determine: (a) the skeleton, i.e., the undirected graph that has the same edges as the DAG but no edge orientations, (b) the v-structures, and (c) the additional edge orientations.

In step (a) the algorithm starts with a complete undirected graph. Then, for each edge (say, between *j* and *k*) the constraint is tested, whether there is any conditioning set, *S* so that *j* and *k* are conditional independent given *S* (i.e., the independence null hypothesis was not rejected at some significance level, *α*). If such a set, called a separation set or *S*(*j*; *k*), is found, the edge between *j* and *k* is removed and the corresponding conditioning set is stored. The algorithm increases the size of the conditioning set step by step, and stops if all adjacency sets in the current graph are smaller than the size of the conditioning set.

In step (b), the algorithm takes into account all unshielded triples, or triples *i* − *j* − *k* where *i* and *k* are not contiguous. The algorithm decides whether or not to align the triple as a v-structure with *i* → *j* ← *k* based on the separating set that causes the removal of *i* − *k*.

In step (c), additional orientation criteria are applied to orient as many of the remaining undirected edges as possible, for more details see [24].

The PC algorithm was shown to be consistent in certain high-dimensional settings [23]. Among all the various modifications of the algorithm, we consider the stable and order-independent version [25].

(2) *Score-based methods.* Score-based methods [6, 7] rely on the fact that each DAG, G∈G may be scored in relation to the data, often using a penalized likelihood score, e.g, the BIC [26]:

$$\begin{array}{c} \hat{G}\in \text{arg}\;\underset{G\in G}{\text{min}}\;\;S\left(G;Y\right)≔-\text{log}L\left(Y;G\right)+\lambda \left|E\right| \end{array}$$ (2)

where *L*(*Y*; *G*) is the likelihood function of the SEM mapped on the DAG *G*, |*E*| represents the number of parameter (edges) in the model, and λ is a penalized parameter (λ = log(*n*) for BIC). The algorithm then looks for a CPDAG that gives the best score. Greedy techniques are often utilized because the space of potential graphs, G, is too large. One of these is the well-known two-phase approach known as Greedy Equivalence Search (GES, [6]). Specifically, by doing a search on the space of potential CPDAGs through the Markov equivalence classes, GES discovers the CPDAG of the underlying causal DAG. In the forward phase of its greedy search, it does single edge additions to maximize score improvement, and in its backward phase, it performs single edge removals. High-dimensional consistency of GES was demonstrated by [9].

(3) *Hybrid methods.* The hybrid methods learn the CPDAG by combining the ideas of constraint-based approach and score-based methods. Among the hybrid approaches, here we consider a novel version of the GES algorithm, called adaptively restricted greedy equivalence search (ARGES), introduced by [9]. ARGES uses a greedy search on a restricted search space using as input the skeleton of the PC algorithm or an estimated conditional independence graph (CIG), i.e. an undirected graph with an edge between *j* and *k* ⇔ cor(*Y*_*j*_; *Y*_*k*_|*rest*) ≠ 0, derived from a preliminary search. It also changes adaptively the forward phase of GES, by restricting edge additions. Let *G* be the loop CPDAG and *j* and *k* be two of its non-adjacent nodes. Then an edge connecting *j* and *k* is acceptable if (i) *j* and *k* are adjacent in the (estimated) skeleton of *G* or (ii) there is a node *v* such that *j* → *v* ← *k* is a v-structure in *G*. At every stage of the algorithm, shields of v-structures (or unshielded triples) in the current CPDAG are allowed in addition to the CIG’s (or CPDAG-skeleton’s) edges. ARGES scales well to sparse graphs with thousands of variables, and as GES, the output is a consistent estimate of the CPDAG.

#### Order-based methods

Exact DAG recovery (without causal faithfulness assumption) has recently received a lot of attention. It has been demonstrated that algorithms based on correct model definition are capable of differentiating between various DAGs in the same equivalence class. This advantage is attributable to more data distributional assumptions than just conditional independence relations. Different studies have emphasized that under certain conditions, such as linearity with constrained error variances, linearity with non-Gaussian errors, and non-linearity with additive errors, unique identification is achievable by topological ordering search.

The topological ordering of the variables (nodes) of a DAG *G* is defined as a non-unique permutation *π* of the nodes: *Y*_1_ ≺ *Y*_2_ ≺ … ≺ *Y*_*p*_, where the relation *k* ≺ *j* is understood to mean that node *k* comes before node *j* (i.e., there is an acyclic route connecting node *k* and node *j*). Formally, *π*_*k*_ < *π*_*j*_ ⇔ *j* ∈ *de*(*k*) and *k* ∈ *an*(*j*), where *de*(*k*) are the descendants of the *k*-th node, and *an*(*j*) are the ancestors of the *j*-th node in the DAG *G*.

These algorithms decompose the DAG learning problem into two phases: (i) Topological order learning under certain conditions; (ii) Graph estimation, depending on the learned topological order, via a step-wise selection procedure of the ancestor nodes.

We present a brief review of the identifiability conditions:

(1) *Linearity with constrained error variances.* According to [14], when the observational data are produced using a Gaussian linear SEM that captures the causal linkages and has equal error variances, the causal graph may be distinguished from the joint distribution. In addition, [27, 28] provide relaxed identifiability conditions with heterogeneous variances, requiring an explicit order among the noise variances. In detail:

- *Equal error variance assumption* [14]: $\text{Cov}\left[U\right]=\text{diag}\left(\sigma_{1}^{2},...,\sigma_{p}^{2}\right)=\sigma^{2}I$;
- *Bottom-up variance assumption* [27]: the noise variance of the child node (variable) is approximately larger that the one of its parents (ancestors), $\sigma_{j}^{2}>\sigma_{k}^{2},j=\pi_{m}\in V,k=an\left(j\right)$;
- *Top-down variance assumption* [28]: the noise variance of the parent node (variable) is approximately lower that the one of its child (descendants), $\sigma_{j}^{2}<\sigma_{k}^{2},j=\pi_{m}\in V,k=de\left(j\right)$.

Along these lines, numerous order-based learning techniques are put forth to determine the precise DAG structure [7, 10, 27, 29, 30].

For example, the top-down algorithm can be specified as follow. Stage (1) infers the ordering by successively finding sources. We start with the set which contains all nodes, *R* = *V* and the empty set, *S* = ∅. We iterate over *R* and *S*: for each node in *R* we calculate its conditional (error) variance given all nodes in *S*. We select the node with the *lowest* variance and append it to the ordering set, *S*, and we also remove it from the remaining set, *R*. With the updated *R* and *S*, we repeat the process of finding the node with the lowest conditional (error) variance given the nodes in *S*, we append it to the ordering set *S* and we remove it from the remaining nodes in *R*, and so on until *R* = ∅. Lastly, the node ordering in *S* is returned. Once the ordering has been estimated, in Stage (2) existing linear (or nonlinear) variable selection methods (glmnet, leaps, L0learn, etc) allow to learn the parent set *pa*(*j*) and hence the DAG *G*. Limitation of this procedure is that can be challenging to actually confirm assumptions of equal or ordered noise variances.

(2) *Linearity with non-Gaussian errors.* Recent research has demonstrated that, without requiring any prior information of the network structure, the application of non-Gaussianity may reveal the whole structure of a linear acyclic model, that is, a causal ordering of variables and the strength of their connections. The linear non Gaussian DAG, often referred to as the linear non-gaussian acyclic model (LiNGAM) [11], relaxes the Gaussianity condition and does not call for an additional constrained noise variance assumption for identifiability. All external unobserved errors, *U* are continuous random variables with non-Gaussian distributions, zero means, non-zero variances, and are independent of each other such that no hidden confounding factors exist.

As shown by [11], the causal ordering of a linear non-Gaussian DAG may be reconstructed via iterative search methods. Specifically, [31] proposes a novel approach, called directLiNGAM, to estimate a causal ordering of variables that ensure the validity of the DAG identification in the LiNGAM model. This procedure calculates the topological (causal) order of variables by sequentially computing residual errors from the model’s input data. It is carried out with a top-down procedure, starting at the root nodes, followed by the children of the root nodes and so on until completion. In detail:

- (a) Given the observed data matrix *Y* and the order list *π* = ∅, perform linear regressions of *Y*_*j*_ on *Y*_*k*_ and compute the residual vectors, $R_{j}^{\left(k\right)}=Y_{j}-{\hat{\beta }}_{jk}Y_{k}$ for all (*j* ≠ *k*) ∈ *V*/*π*. Then, the root node, *Y*_(1)_ in the order list, i.e. *π* = *Y*_(1)_, is identified as the most independent variable: $Y_{\left(1\right)}={\text{min}}_{k\in V/\pi }\sum_{j\neq k}\text{IND}\left(Y_{k};R_{j}^{\left(k\right)}\right)$, where IND is a non-parametric independence test;
- (b) collect the (*p* − 1) residuals of the root node in a new data matrix, *R*^(1)^, i.e., removing the effect of the root node, perform step (a) on these residuals, and append the new root *Y*_(2)_ in the order list, *π* = (*Y*_(1)_, *Y*_(2)_);
- (c) repeat (a)-(b) until *R*^(*p*−1)^ = ∅.

To note, non-Gaussian errors are crucial because, for a Gaussian random variable, uncorrelated and independent are equivalent, so the residual are always independent of its regressors. Vice versa, when the errors are non-Gaussian, the independence of residuals and regressors can be used to select the root sequence with the independence (IND) measure.

Once the causal ordering between the variables are established, it is simple to estimate the strength of the relationships of a strictly triangular matrix *B* by following the order in *π*, using a SEM covariance-based procedure such as least squares and maximum likelihood approaches, pruning the non-significant (*P* > 0.05) regression coefficients, or via a top-down nodewise-based model selection procedure of ancestor nodes.

(3) *Non-linearity with additive errors.* Non-linear transformation is frequently used in data generation in practice, hence it should be considered as an alternative to linear models. A functional causal model, called additive noise model (ANM), depicts the causal effect on each *Y*_*j*_ as a function of the direct causes *Y*_*pa*(*j*)_ and some additive unmeasurable noise, *U*_*j*_ [14]:

$$\begin{array}{c} Y_{j}=f_{j}\left(Y_{pa(j)}\right)+U_{j},\;\;\;\;\;\;j\in V \end{array}$$ (3)

where *U*_*j*_(*j* = 1, …, *p*) are (mutually) independent with Gaussian distribution, i.e., there are no hidden variables. The function, *f*_*j*_ is a suitably functional class and describes how the outcome, *Y*_*j*_, is produced from its causes, *Y*_*pa*(*j*)_), supposed independent with noise errors. Since the independence constraint between noise and cause holds only for the correct causal direction and is broken for the incorrect direction, the unique causal structure can be identified.

Several methods have been developed, here we consider the approach in [13], for potentially high-dimensional on a special (and more practical) ANN, or functional SEM, with (mutually) independent and potentially misspecified Guassian errors, called Causal Additive Model (CAM):

$$\begin{array}{c} Y_{j}=\sum_{k\in pa(j)}f_{j,k}\left(Y_{k}\right)+U_{j},\;\;\;\;\;\;j\in V \end{array}$$ (4)

An important results is that if all functions *f*_*j*,*k*_(.) are nonlinear, the underlying DAG structure is identifiable from the observational distribution, *P*(*Y*). An efficient order-based algorithm that can deal with many variables, proposed by [13], consists of three phases (stages). In detail:

- *Preliminary neighborhood selection*. Fit an additive model with a boosting procedure for each variable, *Y*_*j*_ on all the other variables, *Y*_{−*j*}_ for estimating a superset of the skeleton of the underlying DAG with *K* (usually *K* < 10) “possible” parents of *Y*_*j*_;
- *Estimating the topological order by greedy search*. Order search for the variables starts with an empty order and iteratively adds edges between the nodes that corresponds to the largest gain in the negative log-likelihood score, *S*(*G*^*π*^; *Y*). The order search is “restricted” by considering edges compatible with the preliminary neighborhood selection. The graph is completed to a full connected DAG, $G^{\hat{\pi }}$, in which each ordered node *k* has an directed arrow to all *j* if *k* ≺ *j*. $G^{\hat{\pi }}$ corresponds to the best restricted permutation, $\hat{\pi }\left(R\right)$ for the variable indices.
- *DAG pruning by feature selection*. For pruning the full DAG, nodewise additive models can be used by applying significance testing on the covariate functions, usually with a P-value <0.001, or with penalized additive models excluding expected non-parent variables if ${\hat{f}}_{j,k}=0$.

The limitation of the CAM is the heuristic computational complexity of the three stages. In the absence of detailed knowledge of the data generation mechanism, the assumed functional model must be able to capture complex non-linear relationships compared to the simple (and fast) linear model.

#### Gradient-based methods

Aciclicity is the most common assumption in causal discovery and score-based methods uses heuristic greedy algorithms for solving non-convex optimization without feedback loops, i.e., a combinatorial problem that scales super-exponentially with the number of variables. Recent work called NOTEARS [18] provides a new algorithmic framework for score-based learning of DAG models. The procedure is based on a new algebraic characterisation of acyclicity constraint, which recasts the score-based optimization issue as a continuous problem rather than using the conventional combinatorial technique.

In the linear situation, the matrix $B\in R^{p\times p}$ properly encodes the graph *G*, i.e., an edge *j* ← *k* in *G* is present if and only if *β*_*kj*_ ≠ 0. The entire problem may be expressed in terms of *B*. Given a score function, *S*(*B*; *Y*) the solution of *B* is defined by optimizing *S*(*B*; *Y*) subject to the continuous constraint, *h*(*B*) = 0::

$$\begin{array}{c} \text{arg}\;\underset{B\in R^{p\times p}}{\text{min}}\;S\left(B;Y\right)\;\;\;\;s.t.\;\;\;h\left(B\right)=0 \end{array}$$ (5)

where *h* is a **non-negative** non-convex differentiable function used to enforce acyclicity in the estimated graph. Some possible score functions include:

- *Least squares-EV*: $\sum_{j=1}^{p}\left|\right|Y_{j}-\sum \beta_{jk}Y_{k}{\left|\right|}_{2}^{2}$ for linear SEM with equal error variances [14];
- *Negative log-likelihood-EV*: $\frac{p}{2}\text{log}\sum_{j=1}^{p}\left|\right|Y_{j}-\sum \beta_{jk}Y_{k}{\left|\right|}_{2}^{2}$ for linear SEM with Gaussian equal error variances [32];
- *Negative log-likelihood-NV*: $\frac{1}{2}\sum_{j=1}^{p}\text{log}||Y_{j}-\sum \beta_{jk}Y_{k}{\left|\right|}_{2}^{2}$ for linear SEM with Gaussian not-equal errors variances [32].

The function *h* quantifies the “DAG-ness” of the graph, and nowadays the literature contains many different proposals:

- *The NOTEARS condition* [18]. The first differentiable aciclicity characterization of a DAG: *h*(*B*) = tr[exp(*B* ∘ *B*)] − *p*;
- *A polynomial condition* [33]. Proposed to ease the coding effort as the matrix exponential, may not be available in all deep learning platforms: *h*(*B*) = tr[*I* − (*B* ∘ *B*)/*p*]^*p*^ − *p*;
- *The DAGMA condition* [34]. For a non-negative matrix with spectral radius less than one that has better gradients and runs faster than exponential and polynomial conditions: *h*(*B*) = −log det[*I* − (*B* ∘ *B*)].

Where ∘ denotes the Hadamard product, ${\left[B\circ B\right]}_{jk}=\beta_{jk}^{2}$. Usually, the score function includes a sparsity (regolarized) L1-penalty, followed by a thresholding step of the estimated weighted adjacency matrix using a relatively large cut-off of 0.3.

Continuous optimization methods are pervasive in the field of deep learning, whereby highly parameterized networks are optimized using variations on the well-studied gradient-based solvers [35]. In general, these methods are more global than other approximate greedy or 2-3 stages methods. This is because they update all edges at each step based on the gradient of the score and on the acyclicity constraint, and usually have a faster training time since the optimization run is known to be highly parallelizable on GPU.

This has resulted in the confluence of black-box deep learning approaches, and causal structure discovery based on non-linear SEM with Gaussian errors in Eq 4, i.e., NOTEARS-MLP, GraNDAG, DAG-GNN, MCSL, and many others proposal can be found in the recent review [36]. gCastle Python package [37] includes many development gradient-based methods with optional GPU acceleration. In R, the gnlearn package [18] implements linear NOTEARS with least squares EV loss. [38] investigates cases of poor performance of structure learning with continuous optimization.

Table 2 provides a summary of the structure learning methods in terms of the type of algorithm employed, category and output with the main papers for reference. Besides the type of algorithm, these methods differ in three main aspects: (i) the input requirements; (ii) the category; (iii) the output. All the methods require as input a data matrix, *Y*(*n*, *p*) where *n* is the number of subjects and *p* is the number of genes, with the exception of SEMdag that requires also a graph object. The latter can be derived from existing knowledge or can be an empty graph object (if the user decides to implement a full data-driven procedure). Each method represents a different category, in order to provide a comprehensive overview of existing structure learning approaches. Then, PC, GES, and ARGES give as output a CPDAG while the others are able to recover a DAG object. The goal is to find a structure learning method that provides an optimal solution while controlling the computing time of the algorithm.

**Table 2:** Overview of the considered structure learning methods.

Method	Reference	R package	Algorithm	Category	Output
PC	[5]	pcalg	Peter & Clark algorithm	Constraint	CPDAG
GES	[6]	pcalg	Greedy Equivalence Search	Score	CPDAG
ARGES	[9]	pcalg	Adaptively Restricted GES	Hybrid	CPDAG
LiNGAM	[17]	CausalXtreme	Top-down order search	Order	DAG
CAM	[13]	CAM	Greedy order search	Order	DAG
NOTEARS	[18]	gnlearn	NOTEARS (linear) algorithm	Gradient	DAG
SEMdag	[15]	SEMgraph	Bottom-up ordering (TO/TL)	Order	DAG
			Knowledge-based ordering (TO/TL)	Order	DAG

### SEMdag algorithm

Our SEMdag() function uses a two-stage order-based search with prior knowledge-based or data-driven approach, under the assumption that a linear SEM with equal variance error terms is assumed [14]. After determining the vertex (node) or level (layer) order of nodes in stage (1), the DAG may be trained using penalized (L1) regressions in stage (2) [16].

#### Learning ordering

The estimated linear order is determined via a prior graph topological vertex (TO) or level (TL) ordering, or by using a data-driven node or level bottom-up (BU) procedure.

*Knowledge-based ordering.* Topological sorting or ordering of a directed graph’s vertices is only feasible if and only if the knowledge-based graph is a directed acyclic graph, which means we must convert the graph in a DAG. At least one topological ordering exists in every DAG. For DAGs, topological vertex sorting is a linear ordering of the vertices such that vertex *u* occurs before vertex *v* for each directed edge *u* → *v*. We can construct a topological sort with computing time linear to the number of vertices plus the number of edges, i.e., *O*(*V* + *E*). Examples are the Kahn’s algorithm or the Depth First Search algorithm. However, there can be more than one topological sorting for a DAG. To overcome this issue, we consider DAG topological layer (level) sorting.

Given a DAG *G*, define a collection of sets as follows (cf. [30]): *L*_0_, denotes the set of the root (source) nodes in the top layer, $L_{j}=\cup_{m=0}^{j}L_{m}$ and for *j* > 0, *L*_*j*_ is the set of all the source nodes in the subgraph *G*[*V* − *L*_*j*−1_] formed by removing the nodes in *L*_*j*−1_. So, e.g., *L*_1_ is the set of source nodes in *G* − *L*_0_. This decomposes G into *d* + 1 layers, *L*(*G*) ≔ (*L*_0_; …; *L*_*d*_) where each layer *L*_*j*_ consists of the nodes that are sources in the subgraph *G*[*V* − *L*_*j*−1_], and *L*_*j*_ is an ancestral set for each *j*. The number *d* of “layers” denotes the longest possible distance from some nodes in the DAG to a root node and measures the “depth” of a DAG.

The idea of a topological layer enables us to transform a DAG into a distinct and unique topological structure with (*d* + 1) levels, where a node’s parents must be located in the node’s upper layers and acyclicity is thus naturally ensured [39]. In particular, given a DAG *G*, we derive the topological structure of the DAG by allocating each node to a single layer with an iterative leaf-removal procedure. The *leaf-removal* method is a bottom-up process that eliminates all leaf nodes from the network as well as the edges that are incident on them, at each iteration. Leaf nodes are those that have no outgoing edges. In detail:

- Starting with a DAG, the algorithm first creates the transpose of the DAG by flipping the orientation of the DAG’s edges;
- The iterative leaf-removal procedure is applied on the DAG and the transpose of the DAG. The leaf nodes are stored in layers step by step, and the algorithm ends when the network is entirely deconstructed;
- The leaf nodes of the DAG and the transpose of the DAG (identical to the top-down order of nodes in the DAG) are placed in the last and first layers (*L*_*d*_, *L*_0_), respectively;
- The topological ordering of the network’s nodes is ultimately determined by reversing the bottom-up ordering of the nodes in the transpose of the DAG and combining it with the bottom-up ordering of the nodes in DAG.

**Fig 1:**
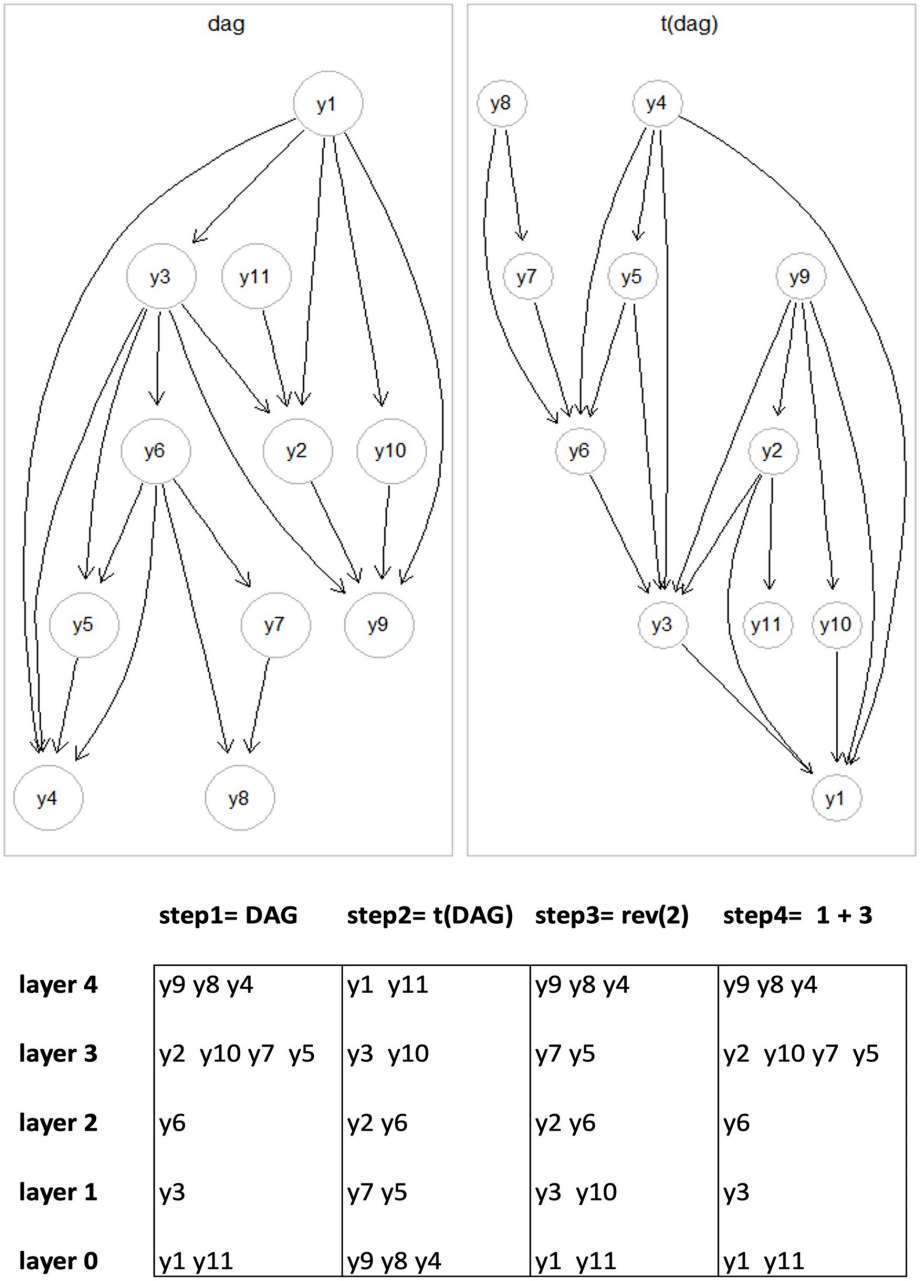
Application of the leaf-removal algorithm on the network of interest (dag) and its transpose (t(dag)) in step(1) e (2) to determine the layers ordering of the network’s nodes by the combination in step(4) of the node structure in step(1) and (3).

We refer the reader to Fig 1 for an example about the leaf-removal algorithm.

Any node, *j* ∈ *L*_*j*_ has some parents in the previous layer, *L*_*j*−1_ and some child in the next layer, *L*_*j*+1_. Learning *G* is equivalent to learning the sets *L*(*G*) = (*L*_0_; …; *L*_*d*_), since any topological sort *π* of *G* can be determined from *L*(*G*), and from any sort *π*, the graph *G* can be recovered via variable selection. Unlike a topological sort of *G*, which may not be unique, the layer decomposition *L*(*G*) is always unique.

*Bottom-up ordering.* The proposed algorithm for learning DAG works by constructing the DAG in a bottom-up fashion as in [27], estimating with a backward procedure the inverse covariance matrix, $\hat{\Omega }={\hat{\Sigma }}^{-1}$ of the sample covariance (correlation) matrix, *S* ≔ (*Y*^*T*^*Y*)/*n* using the graphical lasso algorithm [40]:

$$\begin{array}{c} \hat{\Omega }\in \text{arg}\;\underset{\Omega ⪰0}{\text{min}}\;\;\;\text{tr}\left(\Omega S\right)-\text{log}\;\text{det}\left(\Omega \right)+\lambda \sum_{j\neq k}\left|\omega_{jk}\right| \end{array}$$ (6)

and define step by step a *reversed* causal ordering recovering the minimum precision: var(*Y*_*j*_|*Y*_{−*j*}_)^−1^, i.e., the maximum full conditional variance, from its diagonal elements. To note, if λ → 0 and *n* > > *p*, then the Maximum Likelihood Estimate (MLE) is given as: $\hat{\Omega }=S^{-1}$.

In detail, starting with the empty set (*P* = ∅), each element of the ordering is approximated through the following steps:

1. Select the node with the *highest* full conditional variance as terminal vertex, *Y*_*p*_, i.e. the vertex with minimum value in the diagonal values of the precision matrix, $\hat{\omega }=\text{min}\;\left(\text{diag}\left(\hat{\Omega }\right)\right)$ or the terminal layer (>1 vertices, *L*_*d*_) with $\hat{\omega }\in \text{min}\;\left(\text{diag}\left(\hat{\Omega }\right)\right)+\eta$. The latter means that all nodes with a maximum distance of *η* from the precision value of the terminal vertex’s can be combined to determine the terminal layer rather than just one terminal vertex.
2. Append *Y*_*p*_ or *L*_*d*_ to the ordering set, *P* and also remove the selected node(s) from the column(s) of the data matrix. With the updated data matrix repeat the process of estimating the precision matrix and identify the vertex (or vertices) with the lowest precision(s);
3. Repeat (1) and (2) until the source node, *Y*_1_ or the top layer, *L*_0_ is found.

Lastly, the reverse of the node (or level) ordering in the set *P* is returned. For the glasso procedure, we use the penalized parameter, λ = 0.001 or $\lambda =\sqrt{log(p)/n}$ for low (*n* > *p*) or high (*n* < *p*) dimensional data, respectively.

#### Learning parents

After finding the topological vertex (node) or level (layer) ordering, the challenge of estimating the DAG structure (edge set), as stated by [16], may be viewed in terms of penalized likelihood. Assuming as fixed the node or layer ordering from stage (1): *Y*_1_ ≺ *Y*_2_ ≺ … ≺ *Y*_*p*_, or *L*_0_ ≺ *L*_1_ ≺ … ≺ *L*_*d*_, the stage (2) executes parent estimations by doing LASSO (Least Absolute Shrinkage and Selection Operator) regressions of the *j*-th outcome variable on the predictor (ancestor) variables, *S*_*j*_ ≔ {*Y*_*k*_:*Y*_*k*_ ≺ *Y*_*j*_} or *S*_*j*_ ≔ {*Y*_*k*_:*L*_*k*_ ≺ *L*_*j*_} in the vertex or level order list:

$$\begin{array}{c} \hat{\beta }\in \text{arg}\;\underset{\beta \;\in \;R^{k≺j}}{\text{min}}\;\;||Y_{j}-\sum_{k≺j}\beta_{jk}Y_{k}{\left|\right|}_{2}^{2}+\lambda_{j}\sum_{k≺j}w_{jk}\left|\beta_{jk}\right| \end{array}$$ (7)

It is possible to estimate the DAG adjacency matrix, $\hat{A}$ removing (nodewise) the beta coefficients equal zero (*A*_*jk*_ = 0 if ${\hat{\beta }}_{jk}=0$ and 1 otherwise) or using a threshold on the beta absolute values. To allow for differential shrinkage, various penalty factors *w*_*jk*_ might be given to each beta coefficient. There is no shrinkage if *w*_*jk*_ = 0 for some variables, and those variables are always included in the chosen model. If the input graph is known (knowledge-based approach), weights can be based on the graph edges: 0 (i.e., edge present) and 1 (i.e., edge absent).

The λ_*j*_ parameter for each outcome variable in the LASSO regression is chosen by tuning a vector of λ values, or by cross-validation (*p* ≤ 100) or BIC-based (*p* > 100) lambdas selection. To further improve efficiency, some tuning-free schemes (such as λ = (N(0,1)-quantile at *α*/[2*p*(*j* − 1)]) / $\sqrt{n}$, suggested in [16], or $\lambda =\sqrt{log(p)/n}$, suggested in [41] for graphical lasso) can also be enabled.

#### User interface

The example code of the function SEMdag() is as follows.


SEMdag(graph, data, LO = “TO”,
       beta = 0, eta = NULL, lambdas = NA,
       penalty = TRUE, verbose = FALSE, …)


The inputs are: an igraph object (*graph*) that can be a priori graph topological order or a graph with no edges (data-driven procedure: note that in this case it can be created with the function make_empty_graph() of the igraph package, specificying the number of nodes *n* as input); a matrix with rows corresponding to the subjects and columns to the graph nodes (*data*); the linear order method (*LO*, default = “TO”); the minimum absolute LASSO beta coefficient for a new direct link to be retained in the final model (*beta*, default = 0); the minimum fixed eta threshold for glasso bottom-up search (*eta*, default = 0.05); a vector of regularization LASSO lambda values (*lambdas*, default = NA); penalty factors for differential shrinkage (*penalty*, default = TRUE).

Using a two-step order search methodology, the recovered DAG is approximated. Following the determination of the vertex (node) or level (layer) order of p nodes obtained with the glasso() function of the **glasso** R package [42] in step 1), the DAG may be trained using penalized (L1) regressions with the glmnet() function of the **glmnet** R package [43] in step 2).

When choosing between node or layer ordering, the user has to keep in mind the reduced computational burden in the layer-based approached compared to the node one. In detail, in step 1), the layer approach has to identify the order of *d* + 1 layers, where *d* represents the “depth” of the DAG, instead the node approach needs to find the order of *p* + 1 nodes, where *p* is the number of nodes in the DAG. As a result, an high dimensional graph could impact the computation time of the latter step in the nodewise approach. Same consideration could be done for the step 2) where the number of L1 regressions in the nodewise approach is equal to (*p* − 1); instead, for the layer-based one, the number of regressions is equal to *p*—(number of layers), a smaller set compared to the latter.

The output of SEMgdag() is represented by a list containing four objects: *dag*, the estimated DAG; *dag*.*new*, new estimated connections; *dag*.*old*, connections preserved from the input graph; *LO*, the estimated vertex ordering.

To read more about SEMdag() function, in terms of description, usage, function arguments and value, see help documentation: ?SEMdag or refer to https://rdrr.io/cran/SEMgraph/man/SEMdag.html.

## Experimental design

### Benchmark data

For each specific disease, two different datasets have been selected: one for the training process and the other for testing the proposed modelling scheme. Before selecting the data, we’ve checked that each pair of datasets had the same study type (expression profiling by high throughput sequencing, i.e. RNA-seq data), the same platform and a similar number of subjects. This selection procedure resulted in 4 × 2 datasets as shown in Table 3.

**Table 3:** Description of the selected training/testing datasets for each disease.

Data	Type	Split	GSE	n	case	control	p	KEGG pathway	vcount	ecount
ALS	RNA-seq	Train	GSE124439	160	139	21	100	Amyotrophic lateral sclerosis	190	261
		Test	GSE153960	273	206	67	100			
BRCA	RNA-seq	Train	TCGA	224	112	112	100	Breast cancer	133	483
		Test	GSE81538 + GSE205725	377	190	187	100			
COVID-19	RNA-seq	Train	GSE157103	126	100	26	100	Coronavirus disease—COVID-19	54	83
		Test	GSE152641	86	62	24	100			
STEMI	RNA-seq	Train	GSE59867	157	111	46	99	Lipid and atherosclerosis	191	420
		Test	GSE62646	42	28	14	99			

*Amyotrophic Lateral Sclerosis (ALS)*. Amyotrophic lateral sclerosis is a rare kind of neurodegenerative illness that causes the gradual loss of motor neurons that regulate voluntary muscles. For training, we selected postmortem cortex ALS RNA-seq expression data (GSE124439) from [44] with 139 ALS cases and 21 healthy controls. For testing, postmortem cortex RNA-seq data from the NYGC ALS Consortium (GSE153960) were selected, with 206 ALS cases and 67 controls. Network information has been extracted from the KEGG pathway “Amyotrophic lateral sclerosis”, consisting of 364 nodes and 333 edges. For computational purposes, the largest connected component has been retained, corresponding to 190 nodes and 261 edges.

*Breast Cancer (BRCA)*. Breast cancer develops when cells in the breasts multiply and expand out of control, resulting in a mass of tissue known as a tumor. For training, we make use of the (pre-processed) breast cancer RNA-seq dataset from TCGA project [45], which has *n* = 224 human samples, comprising 112 BRCA samples and 112 control samples. For testing, two RNA-seq datasets were combined: GSE81538 [46] for 190 breast cancer cases and GSE205725 [47] for 187 healthy controls. Network information has been extracted from the KEGG pathway “Breast cancer”, consisting of 147 nodes and 488 edges. For computational purposes, the largest connected component has been retained, corresponding to 133 nodes and 483 edges.

*Coronavirus disease (COVID-19)*. The severe acute respiratory syndrome coronavirus 2 (SARS-CoV-2) is the cause of the respiratory infection known as coronavirus disease of 2019 (COVID-19), which is extremely contagious. RNA-seq data from [48] (GSE157103) were considered for training, with a total of *n* = 126 samples with 100 COVID-19 patients and 26 non-COVID-19. Conversely, RNA-seq data (GSE152641, [49]) from whole blood of 62 COVID-19 patients and 24 healthy controls was considered for testing. Network information has been retrieved from the KEGG pathway “Coronavirus disease—COVID-19”, consisting of 232 nodes and 208 edges. For computational purposes, the largest connected component has been retained, corresponding to 54 nodes and 83 edges.

*ST-elevation myocardial infarction (STEMI)*. A heart attack known as a STEMI, happens when an elevation in the ST segment, often results in myocardial injury or necrosis. As training data, we made use of the RNA-seq dataset (GSE59867) from [50] that reports a total of 157 subjects, among which 111 are cases and 46 are healthy controls. As testing data, we selected the RNA-seq dataset (GSE62646) from [51], where 28 subjects were cases and 14 controls. Network information has been extracted from the KEGG pathway “Lipid and atherosclerosis” (a pathway associated with myocardial disease) consisting of 215 nodes and 428 edges. For computational purposes, the largest connected component has been retained, corresponding to 191 nodes and 420 edges.

### DAG structure recovery

**Fig 2:**
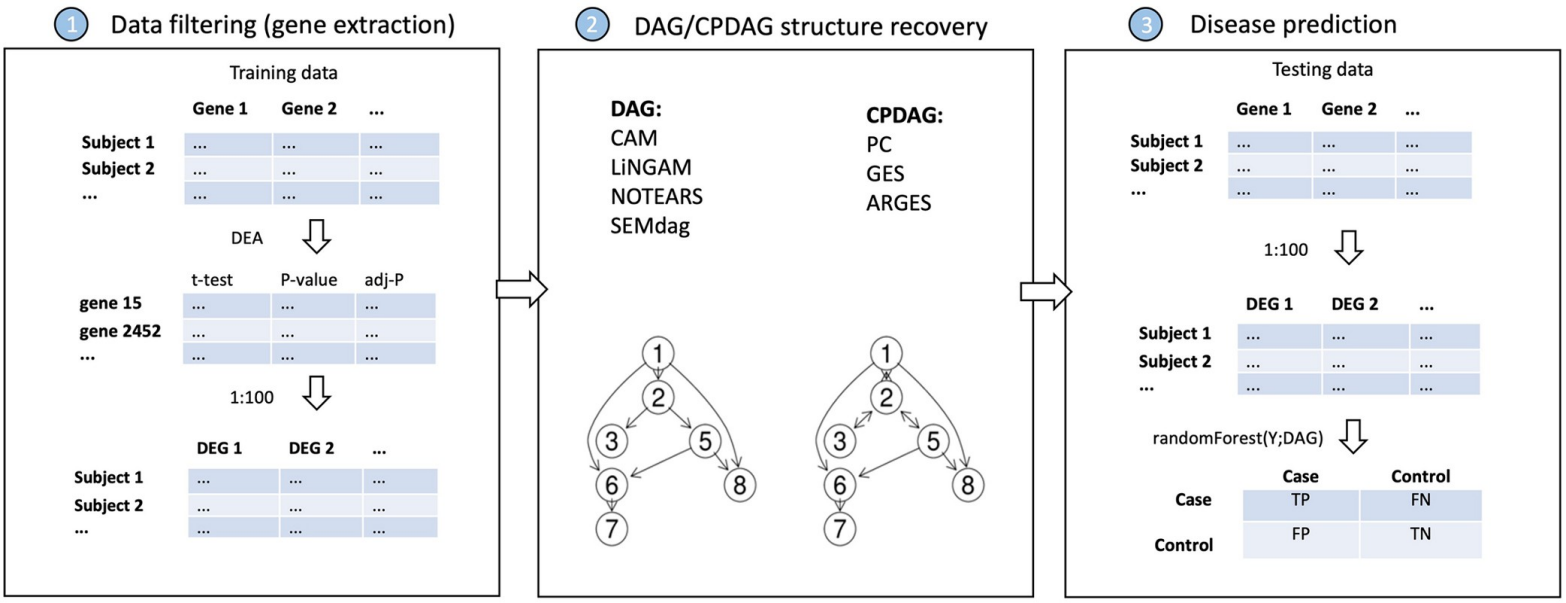
Experimental design scheme.

The causal DAG discovery procedure implemented in this analysis is visually summarised in the first two boxes of Fig 2 and is better explained in this section.

**Fig 3:**
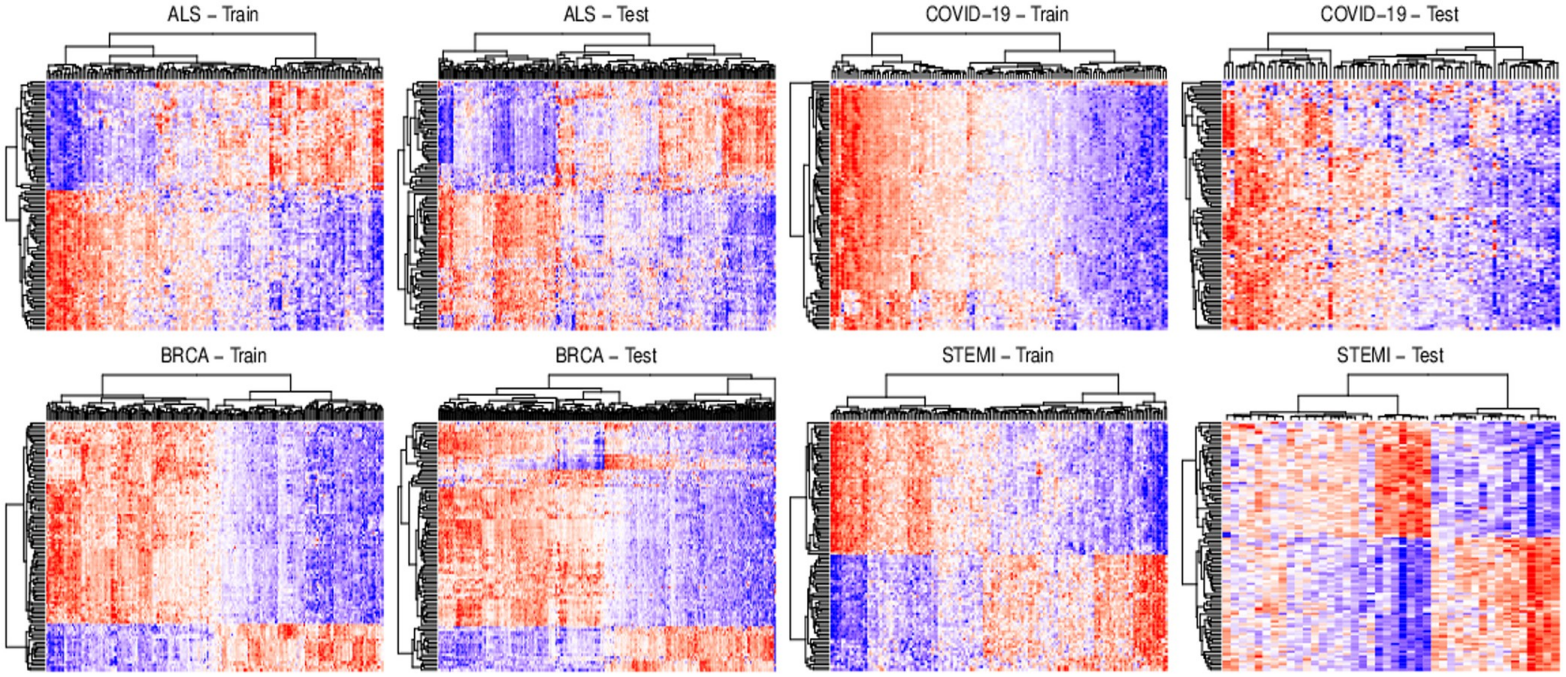
Heatmap of differentially expressed genes (DEGs) for each train/test dataset of each specific disease. The heatmap illustrates expression levels for all DEGs, where red indicates high expression and blue indicates low expression.

- *Data filtering (gene extraction)*. To reduce the computational burden of structure discovery methods, genes of the data matrix have been filtered according to Differential Expression Analysis (DEA). In detail linear models for DEA were fitted with the **limma** R package [52] and p-values were adjusted for multiple testing using the method of Benjamini-Hochberg [53]. In this way, the *p* = 100 most significant Differentially Expressed Genes (DEGs) were filtered out for each dataset, implementing a fully data-driven procedure for causal structure discovery. The differential expression patterns for each pair of datasets of each specific disease is shown in Fig 3. Each pair of datasets share similar differential expression structure, highlighting same biological differences between healthy and diseased states. As a result, the model fitted on the training (learning) data should be well generalizable to the testing (validation) data.
- *DAG/CPDAG structure recovery*. The considered methods run using the default arguments of their R functions, and recover the DAG structure in three different formats:
  (i) adjacency matrix: the functions pc() and ges() from **pcalg** R package [23] estimate the connectivity matrix of a DAG specifying one of various possible methods (PC, GES, or ARGES). On the other side, the **causalXtreme** R package [54] provides wrapper functions for fitting the DirectLiNGAM algorithm and obtaining an adjacency matrix output. In the end, an igraph object has been retrived from the graph_from_adjacency_matrix() function of the **igraph** package [55];
  (ii) edgelist: the function CAM() from the **CAM** R package [13] estimates the edge list of a DAG using the CAM algorithm. From the edgelist output, an igraph object has been obtained from the graph_from_edgelist() function of the **igraph** package;
  (iii) graph: the function notears() from the **gnlearn** R package [56] estimates the DAG structure as an igraph object, without the need for further refinements; the function SEMdag() from the **SEMgraph** package [15] using as argument LO=(BU, TO, or TL) gives as output the igraph object of interest.

All the methods require as input the data matrix, with the exception of SEMdag() that also requires a graph object. So, an empty graph with a number of genes equal to the number of selected DEGs (*p* = 100) is generated and the data-driven bottom-up (BU) search of vertex (or layer) order is performed using the vertices of the empty graph.

### Evaluation metrics

The last step of the experimental design scheme is summarised in the last box of Fig 2. We aim to be able to make out-of-sample disease predictions using the graph structure recovered by the causal discovery algorithms.

In detail, a SEM is fitted for each DAG recovery method, considering the train (learning) disease data of interest together with the graphs of each method. The matrix of the predicted values of train (learning) and test (validation) data are extracted using the path coefficients, ${\hat{Y}}_{train}=Y_{train}\hat{B}$ and ${\hat{Y}}_{test}=Y_{test}\hat{B}$ where $\hat{B}$ represents the MLE of the path coefficients matrix from train data of each CPDAG/DAG model.

Given the predicted data, $\hat{Y}=\left({\hat{Y}}_{train},{\hat{Y}}_{test}\right)$ a Random Forest (RF) approach [57] has been used for making disease predictions for the four (ALS, BRCA, COVID-19, STEMI) disease dataset, being the best performing and most used model in the Machine Learning (ML) framework. It guarantees great predicted precision, adaptability, and immediacy, and is thus regarded as an effective ensemble learning model [58].

RF is performed with the rfCMA() function of the **CMA** R package [59]. In this way, comparisons are made within different causal recovery methods. In addition, RF is also performed on the original data, *Y* = (*Y*_*train*_, *Y*_*test*_) as reference (null graph) benchmark.

Once the disease predictions have been obtained, the aforementioned structure recovery methods have been evaluated with the following metrics:

- MultiDimensional Scaling (MDS): Once obtained the estimated graph structures from each method, the Structural Hamming Distance (SHD) has been computed to generate a measure of structural similarity between graphs by comparing their adjacency matrices. This might be interpreted as how many addition/deletion operations are necessary to transform the edge set of *G*1 into that of *G*2. To obtain a distance measure between 0 and 1, the measurement was related to the size (number of nodes) of each graph; the higher the number, the more distant the objects. Then, a visual representation (MDS) of distances between the obtained SHDs has been generated to identify more or less similar structures (respectively, objects with shorter or longer distances) via the cmdscale() function of the **stats** R package. The graph objects have been divided into *k* clusters, with each observation belonging to the cluster with the closest mean, using the K-means algorithm. The number of cluster (K) is selected via hierarchical clustering (hclust() function of the **stats** R package, with complete linkage method as default). After plotting the dendogram, the optimal height for cutting the tree has been chosen to be the one that better reflects the more distant clusters of objects, joining together the ones with really low SHD values.
- Matthews correlation coefficient (MCC): Out-of-sample predictions for each disease prediction method, categorized as positive and negative cases, have been obtained for testing datasets and compared with ground truth. The confusion matrix, also known as the error or contingency matrix, has been used to assess the diagnostic capacity of classifiers. True positives (TP) and true negatives (TN) are the positive and negative cases that have been correctly identified by the classifier. False positives (FP) are cases where the classifier mistakenly classified a negative as positive, and false negatives (FN) are situations when the classifier mistakenly classified a positive as negative. In binary classification tasks, accuracy and F1 score calculated using confusion matrices continue to be among the most often used measures. However, on unbalanced datasets, these statistical techniques can dangerously show inflated and too optimistic outcomes. Alternatively, a more faithful statistical rate is the MCC, which yields a high score only when the prediction performed well in each of the four confusion matrix categories (TP, FN, TN, FP), proportionately to the size of the dataset’s positive and negative elements (see [60–62] for reference). As a result, DAG structure recovery methods and RF algorithm have been compared with each other using MCC. To note that a MCC = −1 denotes complete disagreement between the prediction and the observation, C = 0 is for a prediction that is no better than random, and C = 1 shows perfect agreement.

To note that, regarding the procedure with SEMdag() function, all four causal structure recovery strategies have been implemented: (i) Knowledge-based ordering (TO/TL) based on the KEGG pathway of the disease of interest, i.e. a biologically validated network structure: *SEMdag*_*KB*_*TO* and *SEMdag*_*KB*_*TL*; (ii) Data-driven Bottom-Up ordering (TO/TL) based on the empty graph with *p* = 100 nodes (DEGs): *SEMdag*_*BU*_*TO* and *SEMdag*_*BU*_*TL*.

## Results

*DAG/CPDAG.* Table 4 reports a descriptive analysis of the recovered graph structures in terms of graph dimension (vertex and edges), number of source and target nodes and measures of centrality as degree and betweenness.

**Table 4:** Descriptive table of recovered DAG/CPDAG structures for each method. V counts the number of Vertices in the network and E the number of Edges; S reports the number of Source nodes and T the number of Targer nodes; D stands for mean(Degree) and B for mean(Betweenness).

	ALS			BRCA			COVID-19			STEMI		
method	G(V,E)	G(S,T)	G(D,B)	G(V,E)	G(S,T)	G(D,B)	G(V,E)	G(S,T)	G(D,B)	G(V,E)	G(S,T)	G(D,B)
ARGES	100;125	3;22	2;58	100;175	1;16	4;124	100;38	62;62	1;0	99;79	21;28	2;8
CAM	100;276	1;15	6;91	100;335	1;9	7;105	100;99	1;43	2;48	99;190	1;17	4;159
GES	100;148	1;5	3;75	100;197	1;5	4;152	100;50	50;50	1;0	99;99	1;1	2;409
LiNGAM	100;436	2;19	9;50	100;471	2;15	9;47	100;470	2;13	9;55	99;503	2;16	10;52
NOTEARS	100;198	6;25	4;58	100;186	6;25	4;72	100;210	4;28	4;26	99;203	3;22	4;82
PC	100;148	18;23	3;13	100;175	25;26	4;8	100;144	29;29	3;3	99;156	29;28	3;4
SEMdag_BU_TL	100;284	2;39	6;11	100;306	2;43	6;10	100;293	3;37	6;9	99;290	6;36	6;8
SEMdag_BU_TO	100;278	1;20	6;45	100;284	1;27	6;60	100;288	2;28	6;40	99;283	2;26	6;36
SEMdag_KB_TL	168;522	12;131	6;8	105;259	28;37	5;19	49;118	10;20	5;8	148;331	27;63	4;27
SEMdag_KB_TO	168;486	3;47	6;80	106;256	3;31	5;21	49;115	3;12	5;16	146;270	14;60	4;49

In terms of node dimension, we have the same number of 100 DEGs for almost all methods except for the STEMI case where we have 99 nodes. Only the *SEMdag*_*KB* methods differ in terms of node dimension, since it depends on the largest component of the KEGG pathway of reference, matched with the nodes in the data. The largest DAGs, with the higher number of nodes together with the most dense structure of connections, are the *SEMdag*_*KB*_*TL* and *SEMdag*_*KB*_*TO* of the ALS and STEMI dataset, where the starting graphs are the largest ones (in terms of nodes) compared to BRCA and COVID-19. After these two methods, the most densely connected graphs are the ones of *LiNGAM* and *SEMdag*_*BU* methods. Lower density graphs are reported by *ARGES*, *GES* and *PC* methods.

It is interesting to understand the number of source and sink nodes reported by each causal discovery method. The highest number of source-sink nodes is reported by *PC* and *SEMdag*_*KB*_*TL* methods for all datasets together with the *ARGES* method for the COVID-19 and STEMI datasets. It can also be pointed out that an high number of sink nodes is reported for all SEMdag() methods. Conversely, the lowest number of source-sink nodes is shown by *GES*.

Degree centrality instead involves counting the number of direct connections a node has; as a result, if high, there is an high number of nodes with high degree (hub nodes). It is interesting to note that the higher mean degree is shown by *LiNGAM*, followed by *SEMdag* methods and the lower one by *ARGES*, *GES*, *PC* and *NOTEARS*.

Betweenness centrality instead involves calculating how often a node occurs on all shortest paths between other pair of nodes. Thus, high betweenness indicate that the structure is characterised by vertices with high influence over the network. Overall, higher betweenness values can be highlighted for *CAM* and *GES* for all datasets and the lower ones for *PC*.

**Fig 4:**
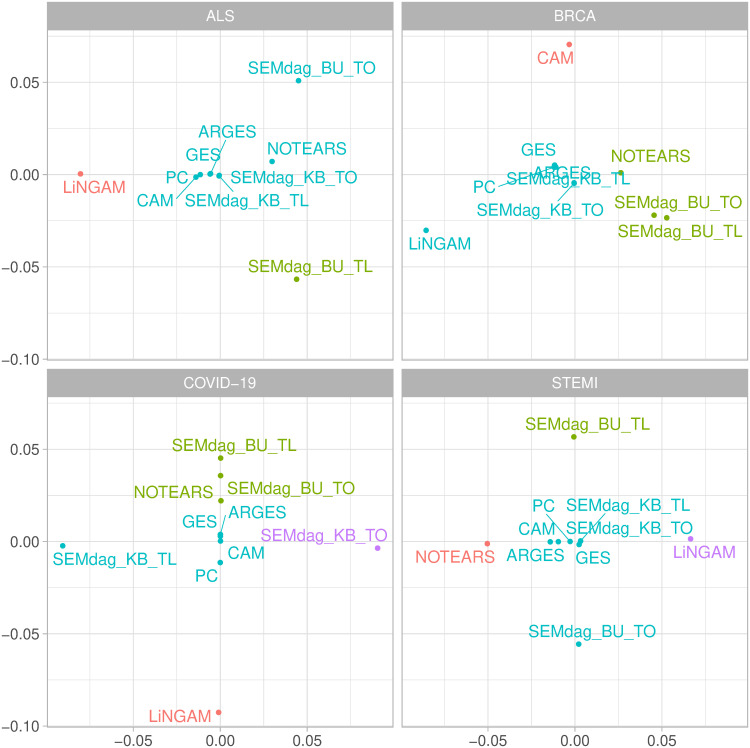
MDS plots across causal discovery methods divided by disease (AD, ALS, BRCA, COVID-19).

*MDS.* Fig 4 shows the MDS plots divided by disease (ALS, BRCA, COVID-19, STEMI). The figures give a quick overview about how the causal discovery methods are grouped together based on the SHD of the recovered graph structures. Generally, a cluster between ARGES-CAM-GES-NOTEARS-PC can be identified, showing similar causal structures (except for BRCA dataset where CAM belongs to a standalone cluster). LiNGAM appears to be distant, in most cases, from all the other clusters, showing a different causal structure. Conversely, SEMdag() methods show a different clustering depending on the dataset of interest: (i) for ALS and STEMI cases, *SEMdag*_*KB* methods are close to each other, belonging to the same cluster together with *SEMdag*_*BU*_*TO*; for BRCA and COVID-19, *SEMdag*_*BU* methods create a cluster with NOTEARS and are distant from *SEMdag*_*KB* methods that belong to different clusters.

**Fig 5:**
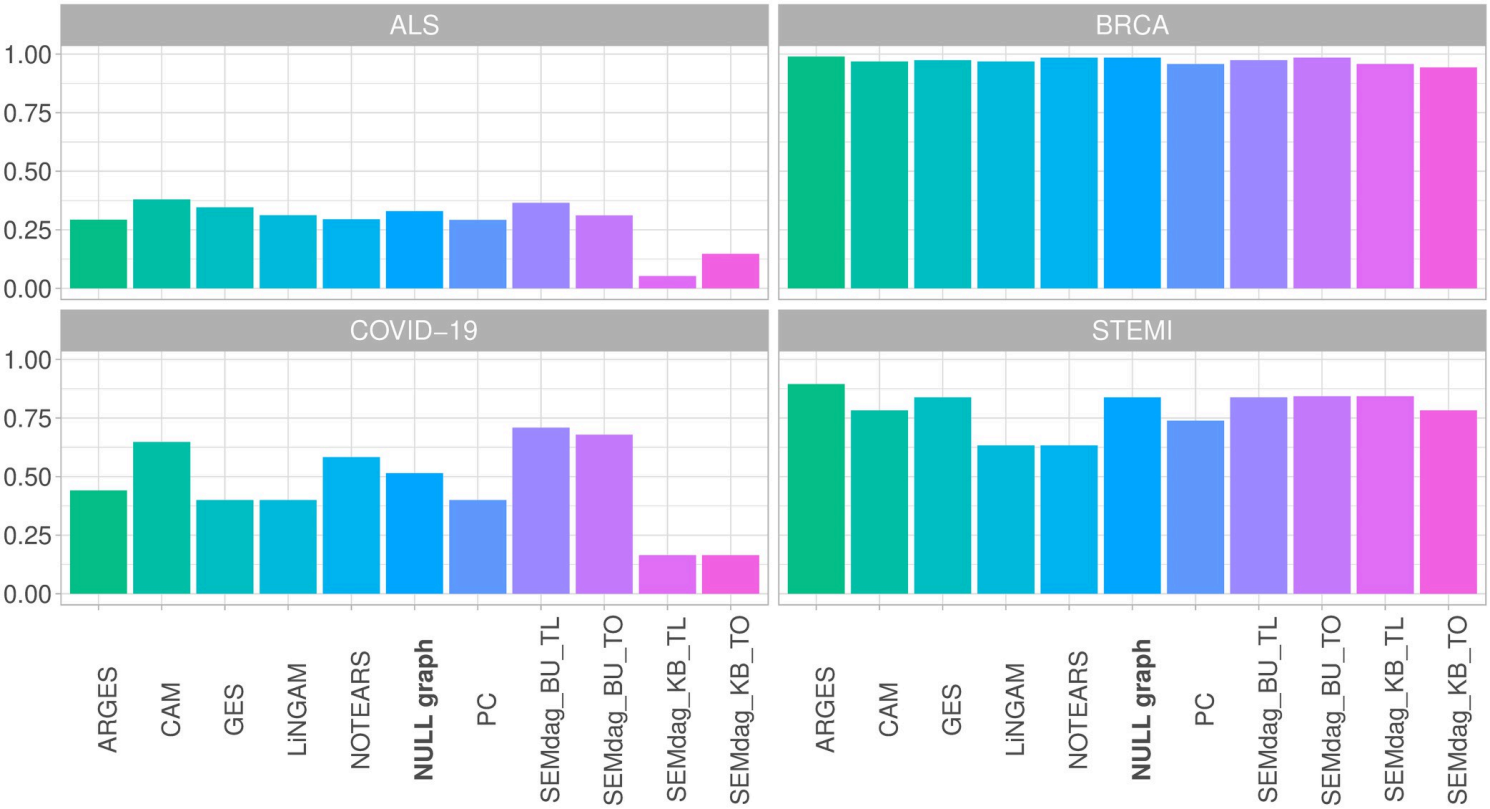
MCC score of RF predictions across causal discovery methods divided by disease (AD, ALS, BRCA, COVID-19).

*MCC.* Fig 5 report the MCC score divided by disease (AD, ALS, BRCA, COVID-19). Overall, higher MCC score is reported for BRCA dataset where MCC reaches almost the level of 1, indicating perfect agreement between the observation and prediction. In the STEMI case, high predictive performance (around 0.75—0.8) is reported by the almost all the methods with the exception of lower metrics showed by *LINGAM* and *NOTEARS*. Regarding COVID-19 data, *SEMdag*_*BU* methods report the highest MCC score around 0.7, exceeding the level of 0.5 reported by most of the methods. To note that the worst score is shown by *SEMdag*_*KB* approaches. Conversely, the ALS case is the worst performing one, with all the performances around 0.3 or below. This result was expected since, as shown in Table 3, in the train dataset, the number of case subjects is almost 7 times higher than control ones, resulting in a really imbalanced data design. However, it can be reported that knowledge based methods have the lower MCC score.

To conclude, it seems that when the reference disease graph is sparse (ALS and COVID-19), the data-driven BU approach of the SEMdag() algorithm works better than the KB one. Thus, in this case, previous knowledge is not needed to reach an higher predictive performance. On the contrary, when the graph is denser (BRCA and STEMI), KB approach is able to recover the BU predictive performance.

Note that fitting an RF approach to original *RF*(*Y*) or predicted $RF(\hat{Y})$ data has almost the same or slightly better MCC performance in BRCA and STEMI prediction. However, for ALS and COVID, the DAG recovery structure improves disease prediction, most notably with *SEMdag*_*BU* methods.

## Discussion

Building on existing literature, we have discussed the problem of learning high-dimensional linear SEMs introducing a two-stage DAG search algorithm. First, (1) the linear order is estimated via a priori graph topological vertex (TO) or level (TL) ordering, or by using a data-driven node or level bottom-up (BU) procedure; then, (2) the DAG is estimated using penalized (L1) regressions.

This methodology stands within the class of order-based methods and assumes equal variance of the error terms. SEMdag() differs from the other methods since it requires a graphical structure as input and makes use of different procedures for learning the ordering.

In the experimental design scheme, we performed a set of experiments on observed expression (or RNA-seq) data considering a pair of training and testing dataset for four different diseases, where the latter has been used for disease predictive performance evaluation. Comparisons have been made within a set of structure discovery methods to find a structure learning method that provide an optimal solution while controlling the computing time of the algorithm.

Based on the results, the SEMdag() algorithm shows the best performance compared to the other methods. In detail, the BU approach is able to reach the highest disease predictive performance when the graph is sparse; instead the KB approach is not able to reach that level of MCC score. Conversely, when the starting graph is more densely connected, the KB approach is able to recover the same predictive performance of the BU methods or exceed it. Unlike SEMdag(), the other methods are case-sensitive, having a lower or higher performance depending on the data matrix given as input, not representing a generally optimal solution.

Finally, it’s important to note that to achieve an effective performance of the SEMdag() algorithm, its inputs need to be properly tuned. Among the various inputs, the user has the ability to adjust two specific arguments:

- *beta* (default = 0): Minimum absolute LASSO beta coefficient for a new direct connection to be included in the final model. To obtain a graph with a sparser graph structure (i.e., fewer connections) with the aim of dealing with high dimensionality issues, the user needs to tune a value > 0 for the *beta* parameter. In general, our experience suggests that values in the range of (0.05, 0.1), produces satisfactory results.
- *eta* (default = 0): Minimum fixed eta threshold for the bottom-up search of graph ordering. By default, *eta* = 0, meaning that the order search is done vertex-wise. If the user wants to set an order search layer-based, the *eta* parameter needs to be tuned, in order to find the layers in the bottom-up procedure. In general, taking sufficiently small value <0.05 works well in practice. Alternatively, with *n* > 100 samples, we suggest to fix *eta* = *NULL*, since the *eta* parameter is estimated adaptively using half of the sample data.

The estimated linear order (node-wise or layer-wise) can be obtained by employing either a priori graph topological (TO) ordering, or a data-driven bottom-up (BU) method. However, for larger graphs or genes, the layer-wise order is the first choice from both a computational and an interpretational perspective.

## Conclusion

We have addressed the issue of learning high-dimensional linear SEMs by improving previous research and presenting a two-stage approach called SEMdag() and included in the R package **SEMgraph**. Using penalized (L1) regressions, the DAG is estimated after first extracting a node (vertex) or layer (level) ordering of the p nodes. The SEMdag() method produces favourable results in terms of MCC metric and, in contrast to existing literature, has the advantage of requiring less computational effort. Furthermore, it permits the user to select different procedures for learning the structure and enables the recovery of a graph structure with predicted data that closely matches a disease of interest.

Further studies might examine alternative approaches to determine the optimal topological order of a DAG. This could be achieved by reducing the distances between vertices in the ordering, taking into account the constraints imposed by the direction of edges. In the future, we intend to develop an algorithm based on the distances between vertices in the topological ordering, with the aim of reducing the complexity of the system represented by the known (or data-driven) DAG, as outlined in reference [63]. Moreover, we will examine the potential of deep neural networks (DNNs) for nonlinear SEM fitting based on DAG layer-wise ordering. This investigation aims to enhance the interpretability of the black-box neural network, particularly with regard to its depth (number of hidden layers) and width (number of hidden nodes at a given layer).
